# TrypsNetDB: An integrated framework for the functional characterization of trypanosomatid proteins

**DOI:** 10.1371/journal.pntd.0005368

**Published:** 2017-02-03

**Authors:** Vahid H. Gazestani, Chun Wai Yip, Najmeh Nikpour, Natasha Berghuis, Reza Salavati

**Affiliations:** Institute of Parasitology, McGill University, Ste. Anne de Bellevue, Quebec, Canada; New York University, UNITED STATES

## Abstract

Trypanosomatid parasites cause serious infections in humans and production losses in livestock. Due to the high divergence from other eukaryotes, such as humans and model organisms, the functional roles of many trypanosomatid proteins cannot be predicted by homology-based methods, rendering a significant portion of their proteins as uncharacterized. Recent technological advances have led to the availability of multiple systematic and genome-wide datasets on trypanosomatid parasites that are informative regarding the biological role(s) of their proteins. Here, we report TrypsNetDB (http://trypsNetDB.org), a web-based resource for the functional annotation of 16 different species/strains of trypanosomatid parasites. The database not only visualizes the network context of the queried protein(s) in an intuitive way but also examines the response of the represented network in more than 50 different biological contexts and its enrichment for various biological terms and pathways, protein sequence signatures, and potential RNA regulatory elements. The interactome core of the database, as of Jan 23, 2017, contains 101,187 interactions among 13,395 trypanosomatid proteins inferred from 97 genome-wide and focused studies on the interactome of these organisms.

## Author summary

Methods to predict protein function based on sequences enable the rapid annotation of newly sequenced genomes. However, as most of these methods rely on homology-based approaches, non-conserved proteins in trypanosomatids remain elusive for annotation, rendering approximately half of the sequenced proteins uncharacterized. In this study, we developed a user friendly integrated database, TrypsNetDB, which fills multiple gaps in the field by depositing the current interactome knowledge on trypanosomatid proteins and combining this information with other available resources accompanied by related statistical analyses. The database allows automatic inter-species mapping of available data to better characterize the queried proteins in the species of interest. The database is built on fast and reliable ASP.Net framework and provides **(i)** a significant increase in the genome-wide functional annotation of trypanosomatid proteins, **(ii)** potential novel targets for therapeutics against trypanosomatids, and **(iii)** a robust methodology that can be adapted for the functional annotation of other non-model organisms.

## Introduction

Trypanosomatid parasites cause life-threatening diseases in humans and major production losses in animals. They pose global threats, and various issues are associated with available drugs against trypanosomatids (including tolerability, cost, and resistance), necessitating the identification of novel essential parasitic-specific pathways/genes as potential drug targets [1]. However, as supported by whole genome sequencing data, it is well known that species of the trypanosomatid family, while showing high similarity in proteomes with one another, are highly diverged from other eukaryotes [2–5]. This makes the annotation transfer of nearly half of their proteome by homology-based approaches from model organisms unreliable [3].

During the past decade, several genome-wide and focused studies have been conducted to functionally characterize trypanosomatid proteins. The construction of global and local protein interaction maps has served as one of the main resources for functional annotation by reflecting the molecular context of proteins in a cell [6–15]. Several experimental techniques exist to identify the interacting partners of proteins that differ in selectivity and sensitivity. Therefore, one major challenge in the study of protein interactions is the ability to distinguish between the correctly associated proteins from confounding elements that are present in the results of these experiments. It is also helpful to know the potential interacting proteins that are missing from the results of an experiment based on previously known knowledge of the species of interest or other related trypanosomatid species. Several databases have been developed to represent the experimentally identified or computationally inferred physical and functional protein interactions [16–21]. Such databases greatly help researchers to interrogate cellular processes and gain a systems level view of the protein(s) of choice. Although it is of critical importance for studies on trypanosomatids, only a limited number of databases cover information on protein interactions of these parasites, and such interactions are mostly predicted by transferring the available data from other eukaryotes, missing most parts of the published data on trypanosomatid species [16].

Another major approach for the functional characterization of proteins stems from recent technological advances that have allowed measuring transcriptome, proteome, and transcript half-life changes in response to environmental changes, different life stages, or cell conditions [8, 22–38]. Moreover, it is possible to gain insights on the function of a protein by gathering information on: 1) its annotation from resources such as gene ontology, KEGG pathways, and the BioCyc database [37, 39]; 2) protein characteristics, such as protein sequence motifs, isoelectric points, molecular weights, and the number of transmembrane domains; 3) the essentiality of gene knock-down on cell survival [33]; and 4) the potential cis-regulatory elements present in the 3′-UTR of the gene and the collective response of genes containing that regulatory element to environmental changes [29]. Currently, the TriTrypDB database is a gene-centric framework devoted to the kinetoplastid parasites and provides extensive information on the queried protein ranging from genomic sequence and position to involved biological pathways and captured responses in previously reported studies [38]. However, in many cases, researchers are interested in knowing the collective response of a list of pre-specified proteins along with their interacting partners according to large-scale studies rather than focusing on one protein. Combining interaction data with enrichment analyses of gene ontology, molecular pathways, gene essentiality, and protein sequence features is the key to perceiving the function of proteins.

Here, we describe TrypsNetDB, a user friendly, integrated database that fills the aforementioned gaps by not only depositing the current interactome knowledge on trypanosomatid proteins but also combining such information with other available resources accompanied with related statistical analyses. Moreover, the database automatically performs inter-species mapping of the available data and provides information to allow for a better characterization of the queried proteins in the species of interest. Finally, based on the built-in features, the database can help researchers with their interactome related experiments by distinguishing the likely binding partners of a protein from confounding elements identified in their experiments and suggesting other potentially interacting proteins that are missing from the list of queried proteins. Built on powerful ASP.Net framework, the database performance is fast and reliable. TrypsNetDB is freely available at trypsNetDB.org.

## Program description and methods

### Overall view of the database

**Fig 1:**
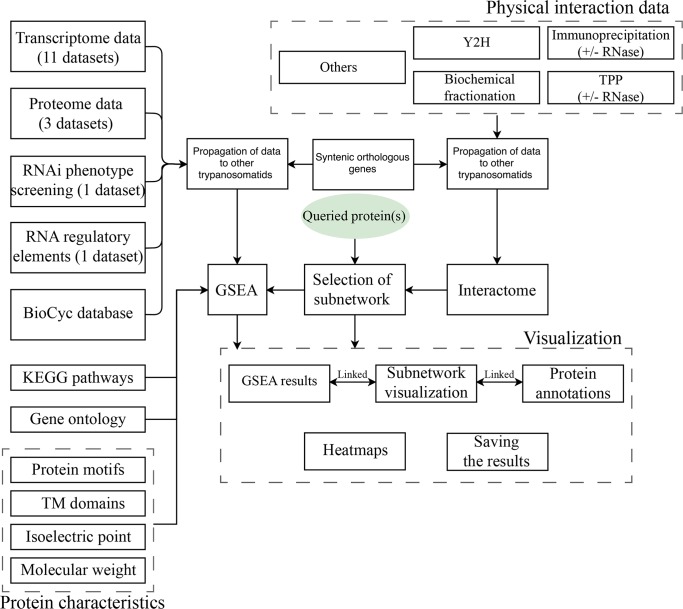
Architecture of TrypsNetDB. The database integrates multiple resources to help functional characterization of trypanosomatid proteins. GSEA: gene set enrichment analysis; TM domains: transmembrane domains; TPP: tagged protein purification.

**Fig 2:**
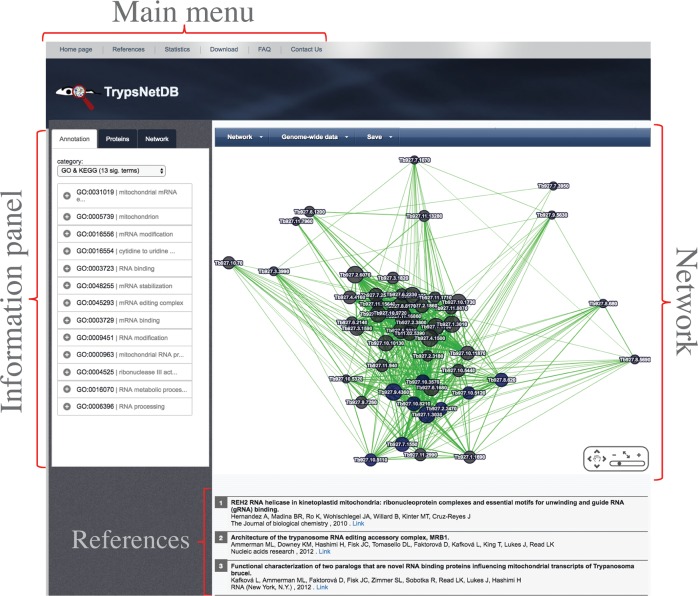
The main result page of TrypsNetDB. The main result page is composed of three elements; that is, information panel, network section, and references section. The information panel contains enrichment analysis results, brief characteristics of the proteins, and the interaction types present in the illustrated network. The network section can be used to explore the interactions among the proteins. Using the menu on the top of the interaction section, users can change the visualization style of the network, access the dynamic heatmaps from various genome-wide data, and save the results. The reference section includes studies from which the interaction data of the illustrated network were extracted with the direct PubMed link.

The current release of the database is focused on physical protein interaction data that are already published in the trypanosomatid field, supporting 16 trypanosomatid parasites including *T. cruzi* strain CL Brener, *T. cruzi* CL Brener Esmeraldo-like, *T. cruzi* CL Brener Non-Esmeraldo-like, *T. brucei* gambiense DAL972, *T. brucei* Lister strain 427, *T. vivax* Y486, *T. evansi* strain STIB 805, *T. brucei* TREU927, *L. major* strain Friedlin, *L. mexicana* MHOM/GT/2001/U1103, *L. infantum* JPCM5, *L. donovani* BPK282A1, *L. braziliensis* MHOM/BR/75/M2903, *L. braziliensis* MHOM/BR/75/M2904, *L. arabica* strain LEM1108, and *L. enriettii* strain LEM3045. Fig 1 represents the schematic architecture of the database. To systematically extract the protein interaction data, we searched the NCBI PubMed database using the keywords *Trypanosoma* and *Leishmania* and extracted all resultant abstracts. Next, by a manual search, initial positive and negative gold standard sets were constructed by considering 46 and 251 articles, respectively. A multinomial naïve Bayes classifier was used to prioritize 6581 articles that were more likely to contain protein interaction data based on the abstract content with an estimated probability greater than 0.75. By a manual inspection of some articles, the initial positive and negative gold standard set was expanded to 60 and 332 articles, respectively (with extra attention on keeping the diversity of the gold standard sets to reduce the chance of biased predictions). The multinomial naïve Bayes classifier was re-trained and then re-applied to all the extracted abstracts from PubMed using the new gold standard set. A total of 1996 articles that were likely to include interaction data were identified (estimated probability of 0.9). By reviewing the articles of the final list, we could extract protein interaction data from 97 different studies. The interaction data were obtained using a variety of techniques, including affinity purification, immunoprecipitation, yeast two-hybrid (Y2H), fractionation patterns, and other possible experimental techniques. We have only considered the syntenic orthologs reported by TriTrypDB to transfer the inter-species information. Users can query the database based on either tritrypDB IDs (recognizing IDs of recent and older versions of the database) or gene names. Support for the remainder of the trypanosomatid species is scheduled to be added in the coming months. In cases where the gene names match multiple organisms, the user will be asked to select the species of interest from a dropdown box. As shown in Fig 2, querying of protein(s) will redirect the user to the interaction page, which is composed of the following three main elements: information panel, network, and reference section.

The information panel can be used to explore the details of the three sections of annotations, protein descriptions, and the constructed network. The annotation tab contains gene set enrichment analyses of the combined set of queried proteins with the suggested proteins by the database (i.e., proteins with gray background) for enrichment in the following five different categories: 1) GO & Pathway: genes are examined for enrichment in Gene Ontology, KEGG pathway, and BioCyc annotations using hypergeometric tests. Terms with Benjamini-Hochberg corrected p-values less than 0.05 are reported back to the user. Hovering over each term will highlight the proteins that are associated with the term. Clicking on each represented term will show a description of the term, its category, number of proteins in the network associated with that term, and the corresponding corrected p-value. 2) Sequence & Structure: The sequence and structural features of the proteins are examined, such as the protein motifs, isoelectric points, molecular weights, and predicted number of transmembrane domains (statistical test for protein motifs is based on the hypergeometric test, and for the other categories based on Wilcoxon-Mann-Whitney rank sum test). This information can provide a complementary view of the function of the proteins. For example, a group of soluble interacting proteins are expected to have a significantly low number of transmembrane domains. Likewise, proteins interacting with RNA and DNA are expected to have high isoelectric points. Similar to the GO and pathway enrichment results, hovering over significantly enriched protein motifs will highlight the associated proteins in the network. 3) Expression patterns: the proteins in the network are examined for their collective transcriptome and proteome responses across 48 distinct samples using Wilcoxon-Mann-Whitney rank sum test. Each sample is color coded with yellow and blue indicating over-expression/enrichment and under-expression/depletion, respectively. Statistically significant terms with p-values less than 0.05 are highlighted by darker colors, while non-significant conditions are semi-transparent. The 48 considered cell states were obtained from genome-wide experiments on *T*. *brucei*, *T*. *cruzi*, and *L*. *infantum* [22–24, 26–28, 30–32, 34–36, 40, 41]. By considering syntenic orthologs (as defined in TriTrypDB), the database automatically propagates the information to other trypanosomatid species. Clicking on each sample will open information on the title of the sample, a description of the results of the statistical tests, the calculated p-value, the title of the study that published the sample and its PMID with a link to the PubMed abstracts. 4) Gene essentiality: The essentiality of proteins in four different cell conditions of *T*. *brucei* are examined based by application of hypergeometric tests on the results of a genome-wide phenotyping study [33]. Ortholog mapping is performed for cases in which the queried organism is not *T*. *brucei*. 5) 3′ regulatory elements: Using a novel approach, we recently predicted 88 cis-regulatory elements that are potentially involved in the developmental regulation of *T*. *brucei* [29]. Although only a limited number of functional elements have been identified thus far, by a rigorous analysis of results, we showed that 11 predicted motifs strikingly resemble previously identified regulatory elements in trypanosomatids, suggesting the high accuracy of the predictions. This section examines whether the 3′-UTRs of the orthologs in the set of proteins in *T*. *brucei* are significantly enriched for any of the predicted 88 motifs using hypergeometric tests. In cases where enrichment is found, the motif logo along with the transcriptome and proteome responses of the motif in different cell conditions are reported.

The proteins tab provides brief information (such as transcript and protein length, isoelectric point, molecular weight, etc.) with a link to the TriTrypDB database in a sorted way, starting from the queried proteins and ending with the proteins that were included in the network by the program (these suggested proteins have been highly connected to the queried proteins based on literature derived interactions).

The network tab can be used to explore the contribution of each experimental technique to the construction of the illustrated network. In two cases of tagged affinity purification and immunoprecipitation in which interactions can show indirect associations, the database distinguishes between interactions that are identified based on RNase treatment of the samples from those that are not. Hovering over each technique will highlight the interactions that they support. It is also possible to filter some of the techniques by unchecking the corresponding checkboxes and clicking on the “set filters” button.

The network section, using a dynamically interactive interface, represents the interactions among the proteins with each protein indicated by a circular node. It is possible to zoom in or out of the network and reposition the proteins. Queried proteins and other proteins suggested by the database are shown in blue and gray, respectively. The node size of proteins indicates the number of interactions that they have in the global network with larger nodes representing nodes with a higher number of interactions. Selecting a protein by clicking on it will highlight the first neighbors of that protein and open the corresponding information in the proteins tab of the information panel. Finally, the network option on the top-left part of the network section can be used to automatically rearrange the network for perhaps a better presentation or to show/hide the protein labels, which may prove useful for the visualization of relatively large networks.

The reference section provides the references from which the interactions were extracted. The full source of resources used for the extraction of the interaction data can be accessed by going to the “References” section from top menu or going directly to the trypsNetDB.org/references.aspx webpage.

### Genome-wide data section

The “Genome-wide data” section on the menu enables users to visualize the genome-wide data available for the queried proteins and their interacting partners suggested by the database. The supported genome-wide data in the current release of the database are categorized in three main groups of fractionation patterns, gene expression patterns, and phenotypic effects, with each containing sub-categories. Users can select one of the main categories (indicated with a blue background) to represent all related sub-categories at once or directly select the sub-categories.

**Fig 3:**
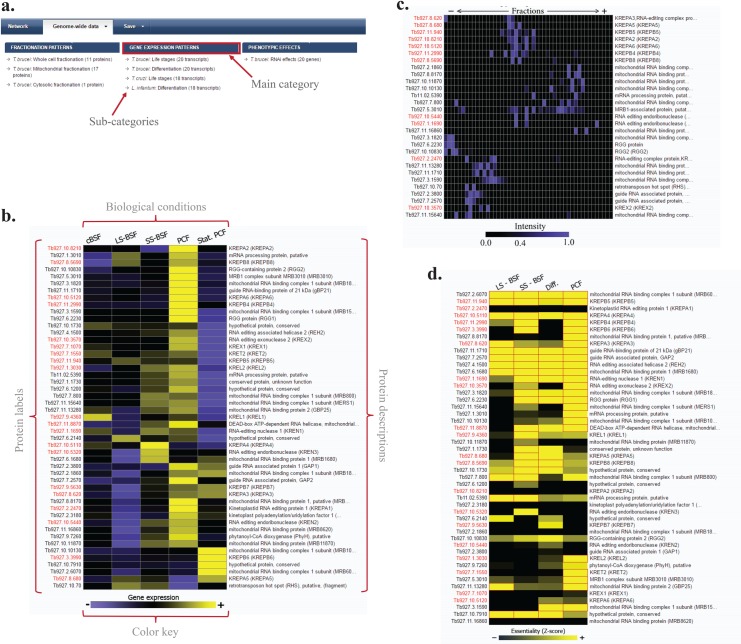
Genome-wide section of TrypsNetDB. (a) Genome-wide sections can be accessed from the menu located on the top of the interaction section. Users can visualize all relevant data by selecting a category or visualize a more specific dataset by choosing the corresponding sub-category. (b) A sample representation of transcriptome heatmap. Queried proteins are highlighted by red labels on the left of the heatmap, while suggested proteins by the database are shown with black labels. (c) A sample representation of a fractionation heatmap. (d) A sample representation of a gene essentiality heatmap. In each life stage (i.e., each column), statistically significant genes are represented by red borders.

This part of the database is particularly useful for the validation of results obtained from interactome-related experiments (such as affinity purifications) by helping users to distinguish between direct binding partners of a protein from potentially spurious elements. For example, the fractionation heatmaps can be exploited to assess whether the potentially interacting proteins show similar fractionation patterns (Fig 3). Currently, the database provides fractionation patterns for whole-cell, mitochondrial-enriched, and cytosolic-enriched cell extracts that can be informative for the localization of previously unannotated proteins (i.e., mitochondrial proteins are expected to be identified in the mitochondrial-enriched fractions while depleted in the cytosolic-enriched sample). Fractionation patterns are also informative regarding the nature of the interactions. As described elsewhere [8], glycerol gradient-based fractionation patterns can capture more transient interactions, while ion exchange-based fractionation favors more stable interactions due to the presence of a salt gradient. Finally, physically interacting proteins are expected to be involved in similar biological processes and, hence, show similar expression patterns and degrees of essentiality in each cell state, which can be easily assessed using the corresponding heatmaps.

### Saving the results

By going to the save option on the top of the network section, users can save the whole represented network or only the sub-networks that are supported by a specific experimental technique. It is also possible to save the enrichment analysis results and the annotations of genes, such as the description, transcript or protein characteristics (length, weight, isoelectric point, and identified SNPs), and gene ontology. Users can also use the save query list option for later regeneration of the same results.

### Implementation

The web application is developed based on the.Net framework 4.5 technology. To improve the performance, the statistical analysis modules (including hypergeometric test, Wilcoxon-Mann-Whitney test, and Benjamini-Hochberg p-value adjustment procedure) were implemented in C# and added as a library to the web application and the performance of the modules has been validated by comparing the results with those of MATLAB 2015b on multiple test sets to ensure accuracy. The network visualization is based on the cytoscape web library, which requires flash player for the representation of the network. All analyses are performed in real-time and a session for each user is ended after 1hr of inactivity. For high-performance, the database is implemented in Microsoft SQL Server 2012.

## Conclusions and future directions

Protein interaction maps remains one of the major resources for the functional annotation of proteins. Embedding other lines of information with these maps can help researchers gain insights regarding the molecular contexts of the proteins. Here, we introduce TrypsNetDB, a web tool to consolidate the current knowledge on the interactome of the trypanosomatid parasites and dynamically integrate them with a wealth of available orthogonal information. We are continuously working on expanding the core, literature-derived, protein interaction depository of the database. Future plans also include providing supports for the remaining trypanosomatid parasites and the inclusion of other genome-wide data. TrypsNetDB is an open source effort, and hence, the code and databases are available through the portal. Moreover, the interaction and fractionation data can be directly downloaded from the web interface using the provided links.
